# Systematic Evaluation for the Influences of the SOX17/Notch Receptor Family Members on Reversing Enzalutamide Resistance in Castration-Resistant Prostate Cancer Cells

**DOI:** 10.3389/fonc.2021.607291

**Published:** 2021-03-10

**Authors:** Zhongbo Du, Luo Li, Wei Sun, Pingyu Zhu, Shulin Cheng, Xuesong Yang, Chunli Luo, Xiaodong Yu, Xiaohou Wu

**Affiliations:** ^1^Department of Clinical Medicine, North Sichuan Medical College, Nanchong, China; ^2^Department of Urology, Affiliated Hospital of North Sichuan Medical College, Nanchong, China; ^3^Department of Urology, First Affiliated Hospital of Chongqing Medical University, Chongqing, China; ^4^Center for Immunology Research, Chongqing Medical University, Chongqing, China; ^5^Department of Urology, Fuling Center Hospital of Chongqing, Chongqing, China; ^6^Key Laboratory of Diagnostics Medicine Designated by the Ministry of Education, Chongqing Medical University, Chongqing, China

**Keywords:** castration-resistant prostate cancer, SOX17, Notch receptor family members, *γ* secretase inhibitors, enzalutamide

## Abstract

The treatment of castration-resistant prostate cancer (CRPC) remains challenging due to the failure of androgen deprivation therapy (ADT); hence the search for other molecular therapeutic targets besides androgen receptor signaling is ongoing. This study systematically investigated the expression of SOX17 and Notch receptors in CRPC tissues and cells *in vitro*, showing that consistent clinical CRPC, SOX17/Notch1, and Notch4 were responsible for enzalutamide resistance in CRPC cells. The *γ* secretase inhibitors, BMS-708163, GSI-IX, PF-3084014, and RO4929097 abrogated the enzalutamide resistance by inhibiting Notch1 or/and Notch4 *in vitro*, with GSI-IX and RO4929097 being more effective than BMS-708163 and PF-3084014 in reliving bone metastasis *in vivo*. In conclusion, the Notch1 and Notch4 inhibitors GSI-IX and RO4929097 are promising therapeutic agents for the treatment of CRPC.

## Introduction

Prostate cancer (PCa) is the second leading cause of cancer-related deaths, accounting for 33,330 deaths in the United States (1). Its mortality rate is also increasing at a rate of 5.5% per year from 2000 to 2011 in China (2). Androgen deprivation therapy (ADT), such as abiraterone, enzalutamide (mainly bicalutamide in China) is the first-line treatment strategy for advanced prostate cancer; however, this relief is temporary and castration-resistant prostate cancer (CRPC) occurs within a few years (3, 4). Chemotherapeutic agents such as docetaxel and cabazitaxel are widely used for CRPC but due to their poor therapeutic effects (5, 6) new-generation drugs, such as abiraterone and apalutamide, have been developed. However, the benefit for CRPC patients is very limited (7–10); hence, the search continues for other molecular targets to treat CRPC.

The signaling pathways Wnt/*β*-catenin (11–14), Hedgehog (15), mTOR/PI3K/AKT (16, 17), and the Notch signaling pathway (18–20) are thought to be involved in the occurrence and development of CRPC. The Notch signaling pathway consists of Notch receptors (Notch1–4), ligands, and downstream target genes (21), and is recognized as an oncogene in various tumors including PCa (22–24). Notch1 signaling is overexpressed in enzalutamide-resistant cells, with inhibition of Notch1 signaling restoring enzalutamide function (25). Also, the downregulation of Notch3 enhances the efficacy of ADT for PCa (20), but there has been no systematic evaluation of the role of the Notch receptors in the drug resistance of CRPC models.

SOX17 (SRY-box containing gene 17) is homologous to the sex-determining gene SRY (26) and a tumor suppressor in various cancers (27–29). On the other hand, SOX17 promotes tumor angiogenesis and destabilizes tumor vessels in Lewis lung cancer, resulting in tumor metastasis and resistance to cisplatin (30). Overexpression of SOX17 initiates and accelerates tumorigenesis (31), but little is known about the role of SOX17 in PCa including CRPC models.

This study investigated the expression of SOX17, Notch receptors 1–4 in prostate cancer and CRPC tissue samples, and enzalutamide-resistant LNCaP cells (Enza-R). Downregulation of Notch receptors was associated with the sensitivity of Enza-R cells to enzalutamide; hence we systematically evaluated the effects of Notch inhibitors on the restoration of enzalutamide sensitivity both *in vitro* and *in vivo*. Some findings of this study may provide a novel treatment approach for patients with CRPC.

## Materials and Methods

### Patient and Tissue Samples

Thirty benign prostatic hyperplasia (BPH) and 36 prostate samples were collected at the First Affiliated Hospital of Chongqing Medical University, Chongqing, China, from September 2018 to April 2019. Thirty-three CRPC tissues, including paraffin and frozen tissue samples, were obtained from the First Affiliated Hospital of Chongqing Medical University, Chongqing, China (25 cases) and the Fuling Central Hospital, Chongqing, China (eight cases) from May 2008 to October 2018. All PCa and CRPC tissues were confirmed by a pathologist. Informed consent was obtained from the patients or their family members and this study was approved by the Ethics Committee of Chongqing Medical University, the Ethics Committee of Fuling Central Hospital, and complied with the Helsinki Declaration.

### Immunohistochemistry

All the embedded PCa and CRPC samples were cut into 5-µm-thick sections. The immunoreactivities of SOX17, Notch1, Notch2, Notch3, Notch4 were detected by an immunoperoxidase staining procedure with the primary antibodies (anti-SOX17, 1:200, Abcam, cat.no.ab192453; anti-Notch1, 1:200, Abcam, cat. no.ab8925; anti-Notch2, 1:200, Cell Signaling Technology, cat.no. D76A6; anti-Notch3, 1:200, Abcam, cat.no. ab23426; anti-Notch4, 1:200, Santa Cruz Biotechnology, cat.no. sc-377399 ). Staining scoring, according to staining intensity, was defined as 0, no staining; 1, weak staining; 2, light staining; 3, moderate staining; and 4, strong staining. Staining scores of ≤1 were defined as negative expression, while staining scores of ≥2 were defined as positive expression.

### Cell Culture

RWPE-1, LNCaP, and DU145 cells were cultured in RPMI-1640 containing 10% fetal bovine serum (FBS) and 1% penicillin/streptomycin. To induce enzalutamide-resistant cells (Enza-R), LNCaP cells were treated with 10 µM enzalutamide (Selleck, USA) for at least 6 months (32), and then 1 × 10^5^ Enza-R cells were seeded into six‐well plates. The lentivirus (Shanghai Gene Pharma Company, China) containing LV‐NC or LV‐shNotch1, LV-shNotch2, LV-shNotch3, and LV-shNotch4, was added to the culture medium for 8 h, then cells were treated with 1 μg/ml puromycin and incubated for 72 h to generate Notch1–4‐silenced stable drug resistance cells.

### CCK8 Assay

The cell counting kit-8 (CCK-8) assay was used to assess cell viability. The cells (2,000 cells/well) were seeded into 96-well plates, then 10 µl CCK-8 reagent (Solarbio, Beijing, China) was added to each well, and the optical density was evaluated using a microplate reader (Bio-Rad Laboratories, CA, USA). For the half-maximal inhibitory concentration (IC50) of enzalutamide, after pretreatment with various agents, such as LV-NC and LV-shNotch1, the LNCaP and Enza-R were transplanted into 96-well plates and cultured with various concentrations of enzalutamide for 24 h using DMSO (Sigma, USA) as control.

### Western Blotting

Protein samples (50 µg) were transferred to PVDF membranes. After blocking with 5% non-fat milk for 2 h at room temperature, the membranes were incubated with primary antibodies overnight at 4°C, anti-E-Cadherin (1:2,000, Cell Signaling Technology, cat.no.14472), anti-N-cadherin (1:2,000, Cell Signaling Technology, cat.no.13116s), anti-Vimentin (1:1,000, Cell Signaling Technology, cat. no.5741s), anti-Zeb-1(1:2,000, Cell Signaling Technology, cat.no.70512s), anti-AR (1:2,000, Cell Signaling Technology, cat.no.19672s), anti-SOX17 (1:2,000, Abcam), anti-Notch1(1:2000, Abcam); anti-Notch2 (1:2,000, CST); anti-Notch3 (1:1,000, Abcam); anti-Notch4 (1:1000, Santa Cruze), and anti-GAPDH (1:1,000, CST, cat. no.5174s) was used as a loading control. The intensity of the protein bands was determined using Image-Pro plus 6.0.

### Immunofluorescence

After cultured with various treatments, the cells were seeded into a 12-well plate and incubated for 24 h, fixed in 4% paraformaldehyde for 20 min, and incubated with various primary antibodies for 1 h in dark room,(anti-Notch1 : 1:00, anti-Notch2: 1:100, anti-Notch3: 1:100, anti-Notch4: 1:100, anti-SOX17:1:150, anti-AR: 1:100). The cells were treated with secondary antibody (Zhongshan Golden Bridge Biotechnology, Beijing, China). DAPI (Zhongshan Golden Bridge Biotechnology) was used for nuclear staining.

### Reverse transcription-quantitative PCR

The Taq™ II kit (Takara, Japan) was used for RT-qPCR on a CFX96™ Real-Time PCR Detection System (Bio-Rad, Hercules, CA, USA). The primer sequences were as follows: Notch1 sense, 5-GAACGGGGCUAACAAAGAUTT-3′, antisense, 5′-AUCUUUGUUAGCCCCGUUCTT-3′; Notch2 sense, 5′-TCAACTGCCAAGCGGATGT-3′; antisense, 5′-CTTGGCTGCTTCATAGCTCC-3′; Notch3 sense, 5′-GCTCAACGGCACTGATCCT-3′, antisense, 5′-AGCCCAGTGTAAGGCTGATT-3′; Notch4 sense, 5′-GGAGACTGC AGACCAGAAGG-3′, antisense, 5′-GACCCTCAGAGTCAGGGACA-3′, AR sense, 5′-TTCCCTCCCTATCTAACCCTC-3′, antisense: 5′-TCTAAACTTCCCGTGGCATAA-3′; SOX17 sense, 5′-ATCCTCAGACTCCTGGGTTT-3′, antisense, 5′-ACTGTTCAAGTGGCAGACAAA-3′.

### Xenograft and Bone Metastasis Model

This study was approved by the Ethics Committee of Chongqing Medical University and performed according to the Guidelines on Animal Experimentation. Enza-R cells (3 × 10^8^) were subcutaneously injected into the left flank or the right tibia of nude mice with surgical castration. After two weeks, the mice with xenograft tumors were treated with enzalutamide and various Notch receptor inhibitors by intraperitoneal injection twice per week. The xenograft tumors in the left flank were evaluated every 5 days. The bone metastasis was harvested after eight weeks, and the xenograft tumors were harvested after four weeks. The tumor-volume (mm^3^) was calculated according to the following formula: volume (mm^3^) = 1/2 × length × width.

### Statistical Analysis

SPSS 19.0 was used for statistical analyses. All data were presented as mean ± SD and analyzed using Kaplan–Meier survival analysis, a stratified log-rank test, Multivariable analyses with the Cox proportional-hazards models, the χ^2^ test, one-way ANOVA, two-way ANOVA, the Student’s t-test, Spearman’s correlation analysis, and the Mann–Whitney test. A P-value <0.05 was considered statistically significant.

## Results

### The Expression of SOX17 and Notch Receptors Was Up-Regulated in CRPC Tissue Samples

**Figure 1:**
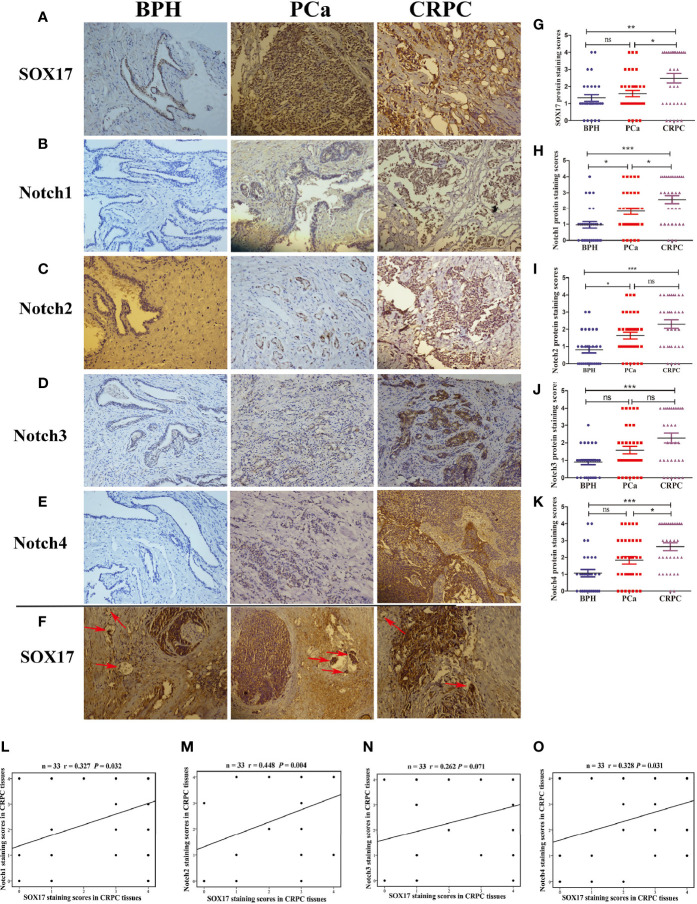
The expression of SOX17 and Notch receptors in samples of benign prostatic hyperplasia (BPH), prostate cancer (PCa) and castration-resistant prostate cancer (CRPC). **(A–E)** The expression levels of SOX17, Notch1 and Notch2, Notch3, Notch4 were detected by using immunohistochemistry (×200). **(F)** The positive expression of SOX17 in cancer thrombus of CRPC (red arrows) (×200). **(G–K)** Average staining scores for SOX17, Notch1, Notch2, Notch3, Notch4 BPH, PPC and CRPC samples. Staining scoring, according to staining intensity, was defined as 0, no staining; 1, weak staining; 2, light staining; 3, moderate staining; and 4, strong staining. Staining scores of ≤1 were defined as negative expression, while staining scores of ≥2 were defined as positive expression. **(L–O)** The correlation curve analysis for SOX17 staining scores *versus* Notch1 and Notch2, Notch3, Notch4 staining scores in CRPC tissues. Values of P < 0.05 were considered to be statistically significant. *P < 0.05, **P < 0.01, ***P < 0.001, ns, no significance.

The expression of SOX17 in CRPC tissues (64%, 21/33) was significantly higher than in prostate cancer tissues (42%, 15/36) (**Figures 1A, G**, **Table 1**). SOX17 was also detected in the tumor thrombus of CRPC suggesting that activated SOX17 may be associated with tumor metastasis (**Figure 1F**). Similarly, the expression of Notch1 and Notch4 was increased in CRPC tissues compared to prostate cancer tissues (**Figures 1B, E, H, K**), but there was no significant difference in the expression of Notch2 and Notch3 (**Figures 1C, D, I, J**). Furthermore, the up-regulation of SOX17 was positively correlated with the expression of Notch1 (r = 0.327, *P* = 0.032) and Notch2 (r = 0.448, *P* = 0.004), Notch4 (r = 0.328, *P* = 0.031) (**Figures 1L–O**).

**Table 1:** Demographic and clinical characteristics of the patients with PCa or CRPC.

	SOX17 expression in PCa			SOX17 expression in CRPC		
	**Negative 21/36 (58%)**	**Positive 15/36 (42%)**	***P*-value**	**Negative 12/33 (36%)**	**Positive 21/33 (64%)**	***P*-value**
Median of PSA (μg/L)	15.31	27.33	**P=0.033**^a^	14.49	23.13	*P*= 0.35^a^
Quartiles 25–75	11.91–28.51	16.09–63.13		10.48–35.02	12.50–33.91	
Gleason score^*^	N = 21	N = 15	*P*=0.09^b^	N = 12	N = 21	***P*=0.006**^b^
≤7	15/21(71%)	5/15(29%)		9/12(75%)	4/21(19%)	
≥8	6/21(29%)	10/15(67%)		3/12(25%)	17/21(81%)	
(New)Bone metastases	15/21(72%)	12/15(33%)	***P*=0.014**^c^	6/12(50%)	12/21(57%)	***P*=0.036**^c^
	**Notch1 expression in PCa**			**Notch1 expression in CRPC**		
	**Negative 16/36 (44%)**	**Positive 20/36 (56%)**	***P*-value**	**Negative 10/33 (30%)**	**Positive 23/33 (70%)**	***P*-value**
Median of PSA (μg/L)	13.83	23.76	*P*=0.052^a^	14.93	23.11	*P* = 0.69^a^
Quartiles 25–75	10.93–30.31	15.55–9.68		11.27–36.93	12.34–33.59	
Gleason score	N = 16	N = 20	***P*=0.01**^b^	N = 10	N = 23	***P*=0.024**^b^
≤7	11/16 (69%)	9/20(45%)		6/10(60%)	7/23(30%)	
≥8	5/16(31%)	11/20(55%)		4/10(40%)	16/23(70%)	
(New)Bone metastases	13/16(81%)	14/20(70%)	*P*=0.51^c^	8/10(80%)	10/23(43%)	*P*=0.602^c^
	**Notch2 expression in PCa**			**Notch2 expression in CRPC**		
	**Negative 18/36 (50%)**	**Positive 18/36 (50%)**	*P*-value	**Negative 12/33 (36%)**	**Positive 21/33 (64%)**	***P*-value**
Median of PSA (μg/L)	16.07	23.76	*P*=0.11^a^	19.21	22.12	*P* = 0.84^a^
Quartiles 25–75	11.46–28.64	15.05–46.77		12.07–35.02	12.21–33.91	
Gleason score	N = 18	N = 18	*P=0.12*^b^	N = 12	N = 21	***P*=0.014**^b^
≤7	7/18(39%)	13/18(72%)		8/12(67%)	5/21(24%)	
≥8	11/18(61%)	5/18(28%)		4/12(33%)	16/21(76%)	
(New)Bone metastases	11/18(61%)	16/18(89%)	*P*=0.53^b^	7/12(58%)	11/21(52%)	*P*=0.586^c^
	**Notch3 expression in PCa**			**Notch3 expression in CRPC**		
	**Negative 21/36 (58%)**	**Positive 15/36 (42%)**	***P*-value**	**Negative 13/33 (39%)**	**Positive 20/33 (61%)**	***P*-value**
Median of PSA (μg/L)	16.77	23.54	*P*=0.52^a^	19.21	15.31	*P* = 0.39^a^
Quartiles 25–75	11.91–36.94	15.31–33.59		12.07–35.02	10.89–29.78	
Gleason score	N = 21	N = 15	*P*=0.096^b^	N = 13	N = 20	***P*=0.009**^b^
≤7	10/21(48%)	10/15(67%)		9/13(69%)	4/20(20%)	
≥8	11/21(52%)	5/15(33%)		4/13(31%)	16/20(80%)	
(New)Bone metastases	16/21(76%)	11/15(73%)	*P*=0.32^c^	8/13(62%)	10/20(50%)	***P*=**0.60^c^
	**Notch4 expression in PCa**			**Notch4 expression in CRPC**		
	**Negative 16/36 (44%)**	**Positive 20/36 (56%)**	***P*-value**	**Negative 8/33 (24%)**	**Positive 25/33 (76%)**	***P*-valu**e
Median of PSA (μg/L)	13.45	23.76	***P*=0.023**^a^	24.00	17.61	*P* = 0.66^a^
Quartiles 25–75	10.36–26.38	16.47–35.02		10.83–40.56	12.22–33.91	
Gleason score	N = 16	N = 20	***P*=0.001**^b^	N = 8	N = 25	***P*=0.003**^b^
≤7	11/16(69%)	9/20(45%)		6/8(75%)	7/25(28%)	
≥8	5/16(31%)	11/20(55%)		2/8(25%)	18/25(72%)	
(New)Bone metastases	11/16(69%)	16/20(80%)	*P*=0.51^c^	5/8(63%)	13/25(52%)	*P*=0.69^c^

PSA, prostate-specific antigen; PCa, prostate cancer; CRPC, castration-resistant prostate cancer. ^a^Mann-Whitney test; ^b^Chi-square test; ^c^McNemer test. Numbers in bold font indicate statistical significance. P<0.05 was confirmed as statistically significant differences. ^*^Gleason’s score was evaluated in both PCa tissues and CRPC tissues, though, in general, there need not Gleason’s score for CRPC.

Next, we determined the association between the expression of SOX17, Notch1, Notch2, Notch3, Notch4, and the key clinical characteristics of PCa and CRPC patients (**Table 1**). The expression of SOX17 and Notch 4 in PCa patients was significantly associated with a higher PSA. Furthermore, Notch1 (*P* = 0.01) and Notch4 (*P* = 0.023) positivity in PCa was associated with a higher Gleason score, while all Notch receptors positive in CRPC were associated with a higher Gleason score. SOX17 up-regulation was associated with bone metastases in both prostate cancer (*P* = 0.014) and CRPC (*P* = 0.036) tissues.

### The Expression of SOX17, Notch1, and Notch2 Was Associated With a Short Progression-Free Survival

**Figure 2:**
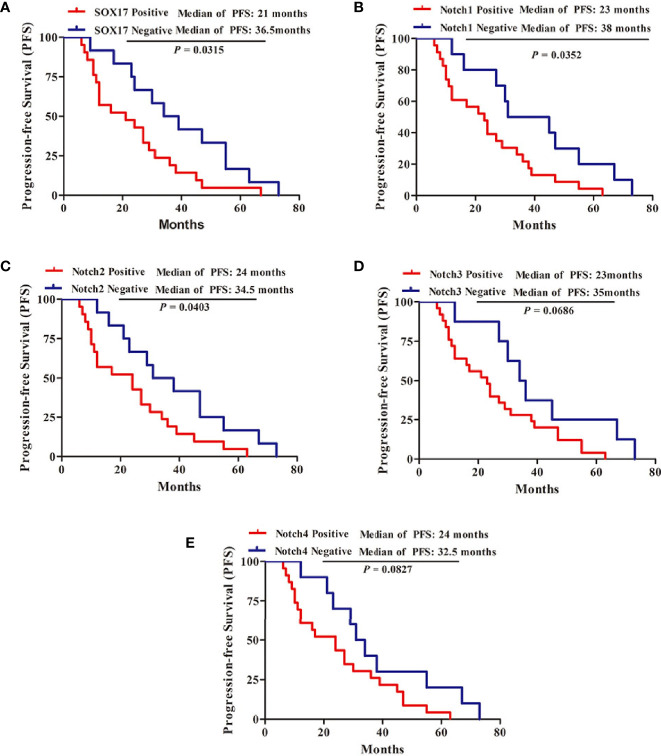
Kaplan-Meier survival analysis for the progression-free survival (PFS) of 33 patients with CRPC. **(A)** 21 patients with SOX17-positive, 12 patients with SOX17-negative, **(B)** 23 patients with Notch1-positive, 10 patients with Notch1-negative, **(C)** 21 patients with Notch2-positive, 12 patients with Notch2-negative, **(D)** 25 patients with Notch3-positive, eight patients with Notch3-negative, **(E)** 23 patients with Notch4-positive, 10 patients with Notch4-negative. P < 0.05 was considered statistically significant.

Kaplan**–**Meier survival analysis was performed to evaluate the relationship between PFS and SOX17, Notch1, Notch2, Notch3, Notch4 in CRPC, revealing that the median PFS was 21 months in CRPC patients positive for SOX17, 36.5 months in SOX17-negative patients (**Figure 2A**, *P* = 0.0315). Moreover, the patients positive for Notch1 had a significantly shorter PFS than Notch1 negative patients (23 *vs* 38 months, *P* = 0.0352) (**Figure 2B**). In addition, the median PFS in Notch2-positive CRPC patients was shorter than Notch2-negative patients (24 *vs* 34.5 months, *P* = 0.0403) (**Figure 2C**). However, the median PFS in patients with Notch3-positive or Notch4-positive was not significantly different with Notch3-negative or Notch4-negative patients (**Figures 2D**, **E**).

Moreover, multivariable analyses with the Cox proportional-hazards models were performed to estimate the simultaneous effects of prognostic factors on PFS of CRPC patients. As shown in **Table 2**, expression of SOX17 (HR = 4.94, *P* = 0.002), Notch1 (HR = 2.85, *P* = 0.044) was linked to a poor prognosis in PFS of CRPC patients (**Table 2**).

**Table 2:** Multivariate Cox analysis for PFS of CRPC patients.

Variables	N	*P* value	HR	95% of CI
SOX17 Positive *vs* Negative	33 21*vs*12 33 23*vs*10 33 21*vs*12 33 20*vs*13 33 25*vs*8	0.002	4.94	1.83–13.34
Notch1 Positive *vs* Negative		0.044	2.85	1.03–7.92
Notch2 Positive *vs* Negative		0.097	2.78	0.83–9.28
Notch3 Positive *vs* Negative		0.263	1.68	0.68–4.15
Notch4 Positive vs Negative		0.553	1.36	0.49–3.78

PFS, progression-free survival; HR, hazards regression; CI, confidence interval; CRPC, castration-resistant prostate cancer.

### The Expression of SOX17 and Notch Receptors Was Up-Regulated in Enza-R Cells, and Knockdown Decreased Enza-R Cell Viability

**Figure 3:**
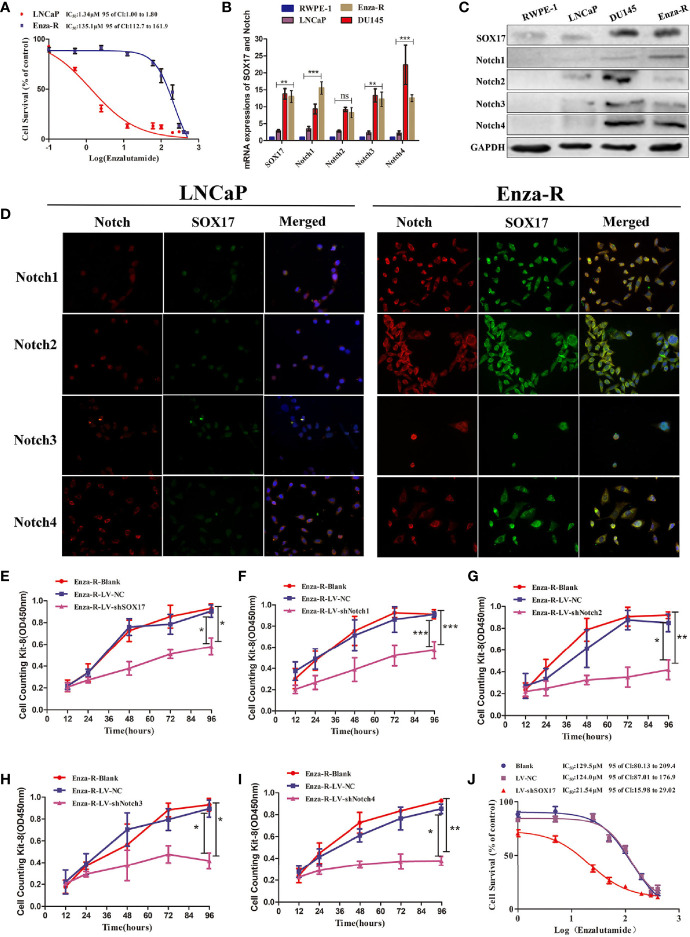
Expression of SOX17 and Notch receptors was detected in Enza-R cells and knocking down them inhibited the viability of Enza-R cells. **(A)** Identification for CRPC cells, both LNCaP and Enza-R cells were treated with increasing concentrations of enzalutamide for 24 h and IC50 was detected by cell counting kit-8 (CCK-8) assay. **(B–D)** Expression of SOX17, Notch receptors in both mRNA and protein level was detected by RT-PCR, Western blot and Immunofluorescence assay (magnification, ×200). **(E–I)** After incubated for 96 h, the viability of Enza-R cells, treated with shSOX17, shNotch1, shNotch2, shNotch3, and shNotch4 respectively, was evaluated by CCK-8 assay. **(J)** Enza-R cells, infected with LV-NC or LV-shSOX17, were treated with increasing concentrations of enzalutamide for 24 h, and the half maximal inhibitory concentration (IC50) was determined by CCK-8 assay. Enza-R: enzalutamide-resistant LNCaP cells. *P < 0.05, **P < 0.01, ***P < 0.001, ns, no significance.

To determine the possible role of SOX17 and Notch receptors in CRPC models, enzalutamide-resistant LNCaP (Enza-R) cells were constructed by continuously treating LNCaP with enzalutamide for at least 6 months. As shown in **Figure 3A**, the enzalutamide resistance of Enza-R cells increased 100-fold compared with LNCaP cells. Also, as expected, the mRNA and protein expression of SOX17, Notch1, Notch3, and Notch4 was up-regulated in Enza-R cells compared with the parental cells (**Figures 3B–D**). To further explore the role of SOX17 and Notch receptors in Enza-R cells, their expression was knocked down using lentivirus, resulting in the significant suppression of cell proliferation (**Figures 3E–I**). Surprisingly, knockdown of SOX17 reversed enzalutamide resistance by nearly six-fold, suggesting that dysregulation of SOX17 is responsible for enzalutamide resistance (**Figure 3J**).

### Knockdown of SOX17 Decreased the Expression of AR and Notch Receptors

**Figure 4:**
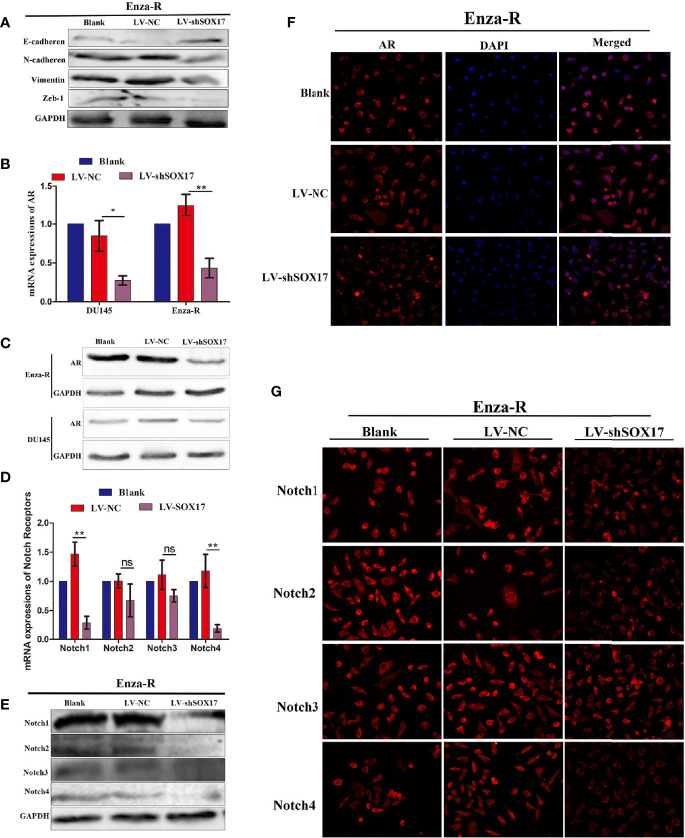
Knocking down SOX17 reducing the expression of EMT related proteins, AR, Notch receptor members. **(A)** Expression of E-cadherin, N-cadherin, Vimentin and Zeb-1 in Enza-R cells, infected with LV-NC or LV-shSOX17, was examined by Western blot assay. GAPDH served as a loading control. **(B–D)** The expression of AR in Enza-R or/and DU145 cells, infected with LV-NC or LV-shSOX17, was evaluated by using RT-PCR, Western blot and Immunofluorescence (magnification, ×200) assay. GAPDH served as a loading control. **(E–G)** Expression of Notch1, Notch2, Notch3, Notch4 were investigated by using RT-PCR, Western blot and Immunofluorescence (magnification, ×200) assay in Enza-R cells treated with LV‐NC or LV‐shSOX17, *P < 0.05, **P < 0.01, ns, no significance; AR, androgen receptor; Enza-R, enzalutamide-resistant LNCaP cells.

SOX17 was expressed in the tumor thrombus of CRPC (**Figure 1F**); thus, it was hypothesized that decreasing SOX17 expression inhibits metastasis. The analysis of the expression of epithelial-mesenchymal transition (EMT) proteins in Enza-R cells revealed that the knockdown of SOX17 was associated with the up-regulation of E-cadherin and the downregulation of N-cadherin, Vimentin, and Zeb-1, indicating that the expression of SOX17 may promote metastasis of CRPC models (**Figure 4A**).

As mentioned above, knockdown of SOX17 restores enzalutamide sensitivity in Enza-R cells. Moreover, it is well known that amplification of AR plays a key role in CRPC; hence, it was hypothesized that SOX17knockdown reverses the resistance by down-regulating AR activity. The mRNA and protein expression of AR decreased in Enza-R and DU145 when SOX17 was knocked down (**Figures 4B–D**). Also, the high expression of SOX17 was positively correlated with Notch1, Notch2, and Notch4 in CRPC tissues (**Figures 1L–O**), so all Notch receptors were detected in SOX17 knockdown Enza-R cells. Reducing SOX17 expression also significantly down-regulated the mRNA expression of Notch1 and Notch4, as well as the protein expression of all Notch receptors, suggesting that SOX17 positively regulates the Notch signaling pathway (**Figures 4E–G**).

### The Knockdown of Notch1 and Notch 4 Partially Restored the Sensitivity of Enza-R Cells to Enzalutamide

**Figure 5:**
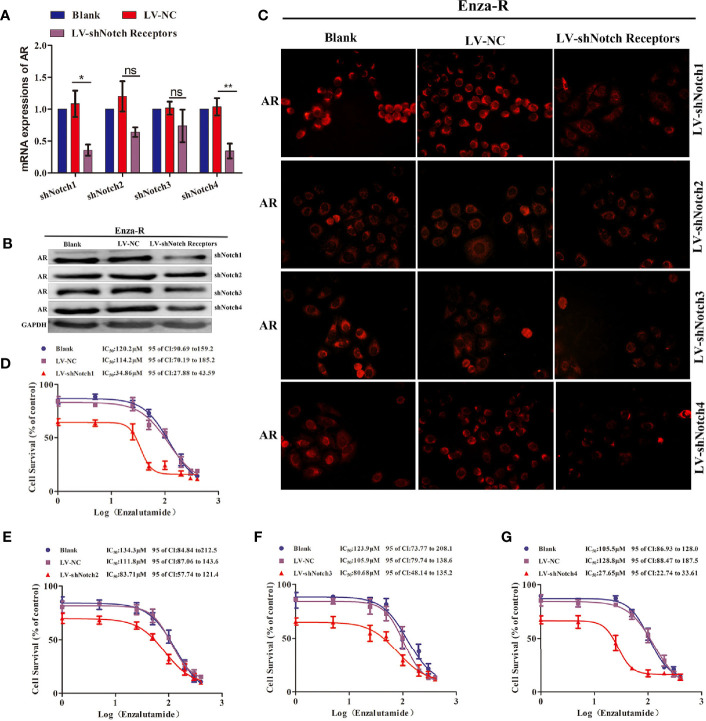
Knocking down Notch receptors inhibited the expression of AR and reversed the enzalutamide resistance in Enza-R cells. **(A–C)** Expression of AR in Enza-R cells infected with LV-shNotch1, LV-shNotch2, LV-shNotch3, LV-shNotch4 were respectively determined by using RT-PCR, Western blot and Immunofluorescence (magnification, ×200) assay. *P < 0.05, **P < 0.01, ns, no significance. **(B–G)** The half maximal inhibitory concentration (IC50) was determined by CCK-8 assay after the cells were treated with increasing concentrations of enzalutamide for 24 h. Enza-R cells were infected with LV-NC or LV-shNotch1, LV-shNotch2, LV-shNotch3, LV-shNotch4 respectively. AR, androgen receptor; Enza-R, enzalutamide-resistant LNCaP cells.

The down-regulation of SOX17 decreased the expression of AR, reversed enzalutamide resistance in Enza-R cells (**Figures 3J**, **4B–D**), and inhibited the expression of Notch receptors (**Figures 4E–G**); thus, it was hypothesized that the down-regulation of Notch receptors also reverses enzalutamide resistance by decreasing AR activity. As shown in **Figures 5A–C**, knockdown of Notch1 and Notch4 significantly inhibited AR expression, however, the inhibition did not be detected in Enza-R cells knocking down Notch2, Notch3. Next, we determined the IC_50_ values of enzalutamide for Enza-R cells knocking down all Notch receptors respectively. Down-regulation of Notch1 and Notch4 increased the sensitivity of the Enza-Rcells to enzalutamide by 3.3-fold, 4.7-fold, respectively (**Figures 5D, G**). These results indicate that dysregulation of Notch1 and Notch4 but not Notch2 and Notch3 is responsible for enzalutamide resistance in Enza-R cells.

### *γ*-Secretase Inhibitors Reversed Enzalutamide Resistance by Decreasing Notch1 and NOTCH4 activity

**Figure 6:**
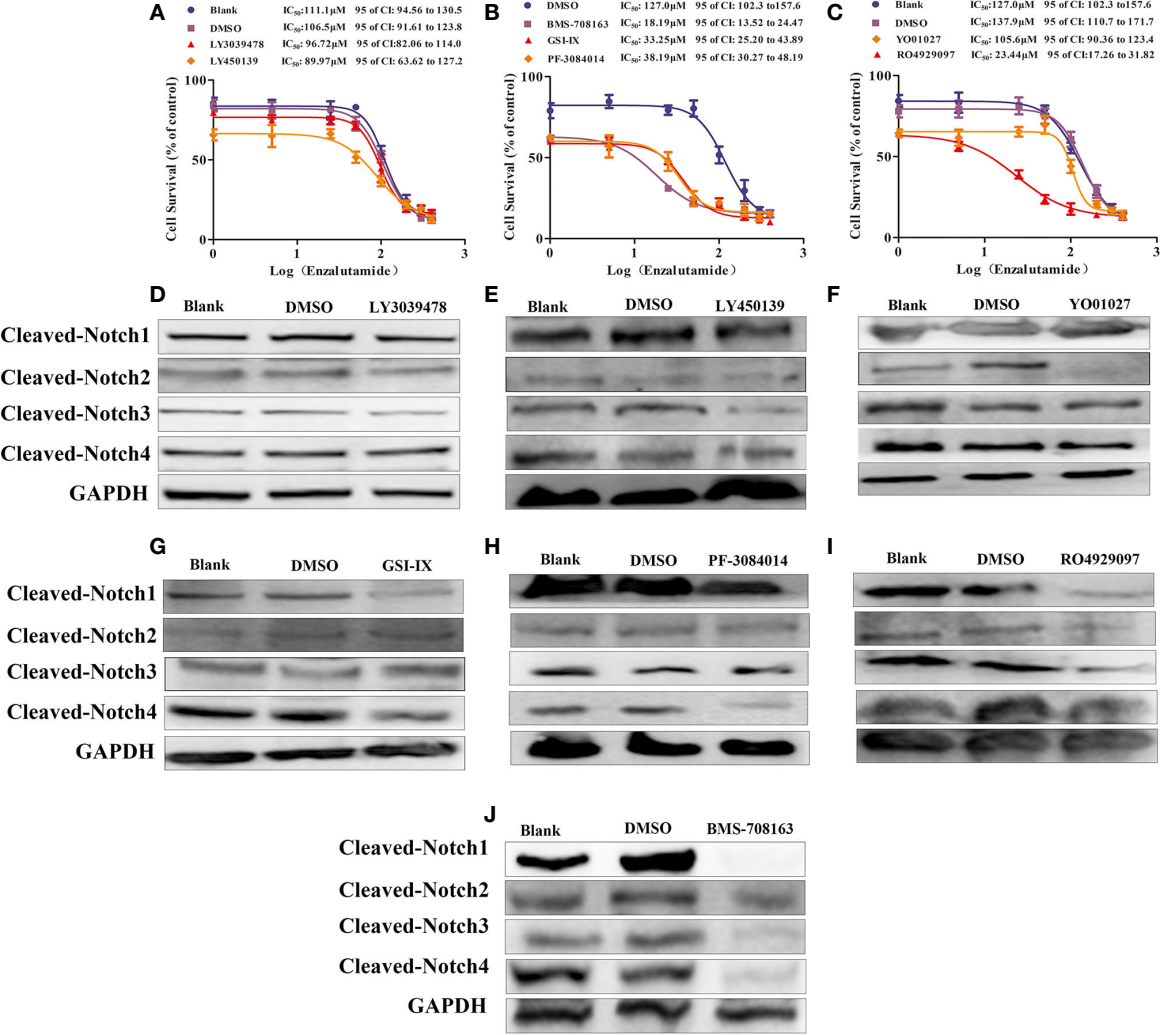
*γ*-secretase inhibitors reversed the resistance by decreasing activities of Notch receptors. **(A–C)** The half maximal inhibitory concentration (IC50) was determined by CCK-8 assay after the Enza-R cells were treated with enzalutamide and *γ*-secretase inhibitors, LY3039478, BMS-708163, LY-450139, GSI-IX, PF-3084014, RO4929097, LY01027 respectively for 24 h. **(D–J)** Activities of each Notch receptor member were determined in Enza-R cells after treated them with various *γ*-secretase inhibitors respectively. The cleaved Notch 1**–**4 means the intracellular domain part of Notch receptor members spliced by *γ*-secretase, which realizes the function of notch receptor members. GAPDH served as a loading control.

The *γ*-secretase complex cleaves Notch receptors into the Notch extracellular domain (NECD) and Notch intracellular domain (NICD), which are transported from the cell membrane to the nucleus and known as “activated Notch” (33–35). It has been reported that γ-secretase inhibitors, such as DAPT and PF-3085014, inhibit malignant biological behavior and reverse ADT-resistance in PCa including CRPC models (18–20, 25, 32). There are many γ-secretase inhibitors on the market, some of which have shown good curative effects on various tumors (36–38); however, their effects on PCa, including CRPC models are still unclear. Moreover, a comprehensive comparison of the effectiveness of these inhibitors on the drug resistance for CRPC models has yet been performed. Thus, seven *γ*-secretase inhibitors were used to decrease Notch signaling (37) in Enza-R cells. Our data revealed that BMS-708163, GSI-IX, PF-3084014, and RO4929097 restored the sensitivity of Enza-R cells to enzalutamide 7.0-fold, 3.8-fold, 3.3-fold, 5.8-fold. However, we failed to find any effects of LY3039478, LY450139, YO01027 on reversing the drug resistance. (**Figures 6A–C**).

PF-3084014 and DAPT enhance the anti-tumor effects of ADT in PCa reversing enzalutamide resistance in CRPC models by decreasing Notch1 activity (18, 25). However, it is still unknown whether these inhibitors reverse the drug resistance by decreasing other Notch receptors besides Notch1. Our data revealed that knockdown of Notch1 and Notch4 but not Notch2 and Notch3 sensitized Enza-R cells to enzalutamide. Also, the BMS-708163, GSI-IX, PF-3084014, and RO4929097 inhibitors affected drug resistance, while others, such as LY3039478 and LY450139 had no effect (**Figures 6A–C**). Thus, we speculated that each *γ*-secretase inhibitor down-regulates one activated Notch receptors, some of which are responsible for the resistance. Western blotting was performed to detect the expression of the activated Notch receptors after the treatment of Enza-R cells with various *γ*-secretase inhibitors. LY3039478, which did not reverse enzalutamide resistance, was unable to decrease any activated Notch receptors in Enza-R cells (**Figure 6D**, **Table 3**). LY450139 down-regulated activated Notch3 but not Notch1, 2, or 4, and failed to reverse enzalutamide resistance (**Figure 6E**, **Table 3**). Although YO01027 inhibited the expression of activated Notch2, it had no therapeutic potential for drug resistance (**Figure 6F**, **Table 3**). Importantly, BMS-708163, by down-regulating activated Notch1, Notch2, Notch3, and Notch4, increased the sensitivity of Enza-R cells to enzalutamide (7.0-fold), GSI-IX, by down-regulating activated Notch1 and Notch4, increased the sensitivity by 3.8-fold, PF-3084014 decreased activated Notch1 and Notch4 restoring the sensitivity of Enza-R by 3.3-fold and RO4929097 inhibited activated Notch1, Notch2, and Notch3 enhancing the sensitivity by 5.8-fold. Taken together, the knockdown of Notch1 or/and Notch4 by lentivirus (**Figures 5E, F**) and down-regulation of activated Notch1 or/and Notch4 by *γ*-secretase inhibitors (**Figures 6G–J**, **Table 3**) restored the sensitivity of Enza-R cells to enzalutamide, further suggesting that over-activated Notch1 and Notch4 are responsible for enzalutamide resistance in Enza-R cells.

**Table 3:** The effectiveness of γ-secretase inhibitors on reversing enzalutamide resistance by inhibiting activities of Notch receptors.

γ-secretase inhibitor	Notch Receptors				Re-sensitive to Enzalutamide(Yes/No)
	Cleaved-Notch1	Cleaved-Notch2	Cleaved-Notch3	Cleaved-Notch4	
LY3039478	−	−	−	−	No
BMS-708163	+	+	+	+	Yes
LY-450139 GSI-IX	− +	− −	+ −	− +	No Yes
PF-3084014	+	−	−	+	Yes
RO4929097	+	+	+	−	Yes
YO01027	−	+	−	−	No

“Cleaved-Notch” means “activated Notch”, the Notch receptors are cleaved into the Notch extracellular domain and Notch intracellular domain (NICD) which is transported from the cell membrane to the nucleus and is called as “activated Notch”. “+” represents γ-secretase inhibitor have effects on Notch receptors, “−” represents γ-secretase inhibitors have no effects on Notch receptors. “Yes” represents γ-secretase inhibitor reversed the enzalutamide-resistant; “No” represent γ-secretase inhibitor fails to reverse the enzalutamide-resistant.

### Combination Therapy of Enzalutamide and *γ*-Secretase Inhibitors Blocks the Growth and Bone Metastasis of Enza-R Cells *In Vivo*

**Figure 7:**
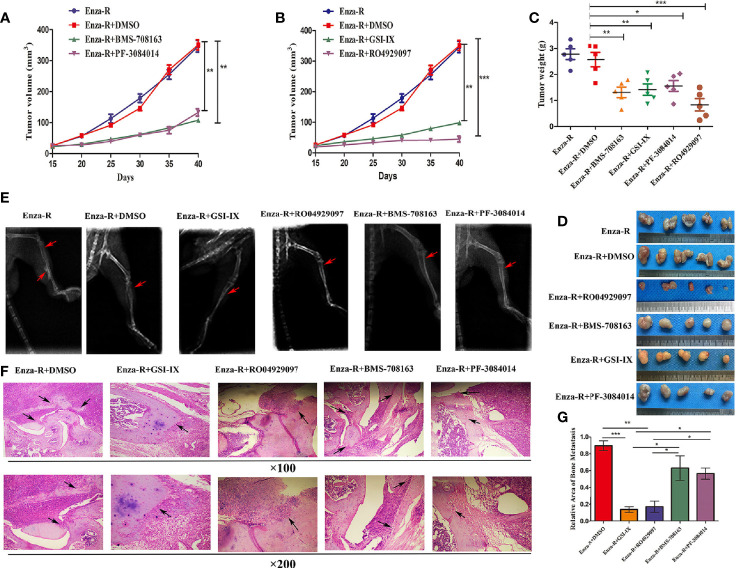
The combination of *γ*-secretase inhibitors and enzalutamide suppressed growth and bone metastasis of Enza-R cells *in vivo*. The nude mice with subcutaneous or bone xenograft were respectively treated 10 mg/kg enzalutamide and 50 mg/kg various *γ*-secretase inhibitors. **(A, B)** Tumor growth curve **(C)** Weight of tumors **(D)** Images of the recovered tumors **(E)** X-ray for bone metastasis **(F)** H&E staining for bone metastasis (upper×100, lower ×200). **(G)** The quantitative results of bone metastasis based on bone metastasis. *P < 0.05, **P < 0.01, ***P < 0.001.

The therapeutic potential of *γ*-secretase inhibitors, BMS-708163, GSI-IX, PF-3084014, and RO4929097, was evaluated *in vivo*. A xenograft tumor was constructed by treating castrated nude mice with a combination of enzalutamide and *γ*-secretase inhibitors. As expected, compared to the control group, BMS-708163, GSI-IX, PF-3084014, and RO4929097 significantly inhibited tumor growth (**Figures 7A–D**). Next, we subcutaneously injected Enza-R cells into the right tibia to investigate the therapeutic potential of the four *γ*-secretase inhibitors for bone metastasis. The mice were treated with a combination of enzalutamide and each *γ*-secretase inhibitor by injection into the tail vein, with X-ray and H&E assays performed to evaluate bone destruction. GSI-IX, RO4929097, BMS-708163, a+nd PF-3084014 relieved the bone damage caused by Enza-R cells (**Figure 7E**). Importantly, our data showed that GSI-IX and RO4929097 were more effective than BMS-708163 and PF-3084014 in reducing bone metastasis (**Figures 7F–G**). This systematic investigation of *γ*-secretase inhibitors indicates that GSI-IX and RO4929097 have the potential for the synergistic treatment of CRPC models with enzalutamide.

## Discussion

SOX17 is a tumor suppressor in various cancer types (28, 39, 40). Of note, recently, some studies revealed that SOX17 may promote rather than suppress tumorigenesis (41, 42). The present study demonstrated that SOX17 is overexpressed in both CRPC tissues and Enza-R cells and associated with a poorer prognosis in CRPC patients. Down-regulation of SOX17 significantly restored enzalutamide sensitivity in Enza-R cells by decreasing AR activity. Taken together, SOX17 is an oncogene in CRPC models, hence may be a potential target for CRPC therapy.

Dysregulation of the Notch signaling pathway is associated with PCa, including CRPC (15, 18–20, 25), with the down-regulation of Notch signaling reducing the growth and invasion of prostate cancer (43–45). The present study showed that knockdown of Notch1 and Notch4 but not Notch2 and Notch3 partly restored the sensitivity to enzalutamide, suggesting that Notch1 and Notch4 are responsible for the drug resistance by inhibiting AR activity. This is in contrast to a study by Yin Wang et al (46) which reported that reducing Notch signaling by down-regulating DLL1 fails to inhibit AR signaling in LNCaP, PC3 cells. In our opinion, unlike in PCa cells, the biological behavior and phenotype of signaling pathways are changed in CRPC models, which may result in an association between Notch signaling and the AR signaling pathway.

Several *γ*-secretase inhibitors have been reported to have anti-tumor effects in various cancer types, for example, clinical trials of PF-03084014, LY900009, and DAPT have shown promising anti-tumor effects in advanced cancer types (47–49). Down-regulation of Notch activities by *γ*-secretase inhibitors, such as DAPT, MK-0752, and PF-3084014, re-sensitizes enzalutamide-resistant prostate cancer cells to enzalutamide (18–20, 25, 32, 45); hence *γ*-secretase inhibitors are promising agents for the treatment of advanced PCa, including CRPC models. In the present study, we found that *γ*-secretase inhibitors significantly re-sensitize Enza-R cells to enzalutamide, with GSI-IX and RO4929097 being more effective than BMS-708163 and PF-3084014 in reliving bone metastasis, suggesting that both GSI-IX and RO4929097 have more potential for the treatment of CRPC by reversing enzalutamide resistance.

Taken together, our findings provide evidence that activity of SOX17/Notch1 or the Notch4 axis is responsible for enzalutamide resistance in CRPC models, and the pharmacological inhibition of the Notch signaling pathway by the *γ*-secretase inhibitors GSI-IX and RO4929097 is a promising therapeutic strategy for CRPC.

## Data Availability Statement

The original contributions presented in the study are included in the article/supplementary material. Further inquiries can be directed to the corresponding authors.

## Ethics Statement

The studies involving human participants were reviewed and approved by the Ethics Committee of Chongqing Medical University. The patients/participants provided their written informed consent to participate in this study. The animal study was reviewed and approved by the Ethics Committee of Chongqing Medical University. Written informed consent was obtained from the individual(s) for the publication of any potentially identifiable images or data included in this article.

## Author Contributions

Conception and design: ZD, LL, CL, XDY, and XW. Development of methodology: ZD, LL, WS, PZ, SC, and XSY. Acquisition of data: ZD, LL, WS, and PZ. Analysis and interpretation of data: ZD, LL, WS, XDY, and XW. Writing, review, and/or revision of the manuscript: ZD, LL, XDY, and XW. All authors contributed to the article and approved the submitted version.

## Funding

This study was supported by the Natural Science Foundation of China (no 81901629), Health and Family Planning Commission of Sichuan Province (no 20PJ154), and Nanchong Municipal Science, Technology and Intellectual Property Office (no. 180180).

## Conflict of Interest

The authors declare that the research was conducted in the absence of any commercial or financial relationships that could be construed as a potential conflict of interest.
